# Quantum Dots Do Not Affect the Behaviour of Mouse Embryonic Stem Cells and Kidney Stem Cells and Are Suitable for Short-Term Tracking

**DOI:** 10.1371/journal.pone.0032650

**Published:** 2012-03-05

**Authors:** Aleksandra Rak-Raszewska, Marco Marcello, Simon Kenny, David Edgar, Violaine Sée, Patricia Murray

**Affiliations:** 1 Institute of Translational Medicine, University of Liverpool, Liverpool, United Kingdom; 2 Centre for Cell Imaging, Institute of Integrative Biology, University of Liverpool, Liverpool, United Kingdom; 3 Department of Paediatric Surgery/Urology, Alder Hey Children's NHS Foundation Trust, Liverpool, United Kingdom; Universidade Federal do Rio de Janeiro, Brazil

## Abstract

Quantum dots (QDs) are small nanocrystals widely used for labelling cells in order to enable cell tracking in complex environments *in vitro*, *ex vivo* and *in vivo*. They present many advantages over traditional fluorescent markers as they are resistant to photobleaching and have narrow emission spectra. Although QDs have been used effectively in cell tracking applications, their suitability has been questioned by reports showing they can affect stem cell behaviour and can be transferred to neighbouring cells. Using a variety of cellular and molecular biology techniques, we have investigated the effect of QDs on the proliferation and differentiation potential of two stem cell types: mouse embryonic stem cells and tissue-specific stem cells derived from mouse kidney. We have also tested if QDs released from living or dead cells can be taken up by neighbouring cells, and we have determined if QDs affect the degree of cell-cell fusion; this information is critical in order to assess the suitability of QDs for stem cell tracking. We show here that QDs have no effect on the viability, proliferation or differentiation potential of the two stem cell types. Furthermore, we show that the extent of transfer of QDs to neighbouring cells is <4%, and that QDs do not increase the degree of cell-cell fusion. However, although the QDs have a high labelling efficiency (>85%), they are rapidly depleted from both stem cell populations. Taken together, our results suggest that QDs are effective cell labelling probes that are suitable for short-term stem cell tracking.

## Introduction

Quantum dots (QDs) are fluorescent semiconductor nanocrystals, which due to their optical properties, have the potential to be used in a variety of biomedical applications [1]. The wavelength of light emitted by the QDs is dependent on size, with larger QDs emitting in the red part of the spectrum, and smaller QDs emitting blue light [1]. QDs have a number of advantages over traditional fluorescent markers, such as green fluorescent protein (GFP). For instance, they have a wide absorbance and narrow emission spectrum, which means that QDs of different sizes can be excited with a single light source and emit at discrete, non-overlapping wavelengths, making them ideally suited to multiplexing [2], [3], [4]. Furthermore, they are resistant to photobleaching [5], which means they have great potential for time-lapse studies. QDs typically comprise an internal CdSe or CdTe core, surrounded by a ZnS shell that stabilises the optical properties of the QDs and prevents the leakage of cytotoxic Cd^2+^ ions [6]. The behaviour of the QDs can be regulated by functionalising the ZnS shell. For instance, to promote cellular uptake, the ZnS can be functionalised with carboxylic acid groups [7] or positively charged peptides [8]. Alternatively, by functionalising the ZnS shell with an appropriate high affinity peptide ligand, the QDs can be targeted to surface receptors of specific cell types [9].

It has been shown previously that QDs can be used to label mesenchymal stem cells (MSCs), allowing the fate of the cells to be tracked *in vivo* following transplantation [10]. QDs are useful for cell tracking because their optical properties enable them to be distinguished from background tissue autofluorescence, which can be a problem when trying to track GFP-labelled cells in adult tissues. A further advantage is that QD labelling is both rapid and highly efficient [11], which is of benefit when labelling some primary cell types that can be difficult to transfect.

However, despite the potential for QDs in cell tracking experiments, a number of contradictory reports have questioned their suitability for such applications. For instance, while some studies have shown that QDs do not affect cell viability [11], [12], others have shown cytotoxic effects [13], and although QDs have been found to effectively label human MSCs without affecting their differentiation potential [10], other reports have demonstrated that QDs inhibit MSCs from undergoing chondrogenesis [14] and osteogenesis [15]. Furthermore, while it has been reported that QDs are not readily transferred to unlabelled host cells [10], it has recently been reported that QDs are excreted from some cell types, and can be transferred efficiently to neighbouring cells [11], [12]; this is obviously a major concern in cell tracking studies as it could lead to false positive results. A difficulty in comparing these contrasting studies is that either different stem cell types were used (MSCs [10] or embryonic stem cells (ESCs) [12]), or in cases where the same stem cell type was used, the QDs had different surface chemistries (carboxyl groups [10] or positively charged peptides [11]), or different techniques to promote QD entry into cells were used (passive uptake [10] or lipofection [15]).

The aim of this work was to investigate the suitability of positively charged QDs for stem cell tracking. To this end, we examined the effect of QDs on the viability, proliferation rate and differentiation potential of two types of stem cells: mouse embryonic stem cells and mouse kidney-derived stem cells (KSCs), a tissue-specific stem cell line isolated from postnatal mouse kidney [16]. We also examined the extent to which QDs are depleted from these stem cells as they proliferate in culture, and determined if QDs released from living or dead cells can be transferred to unlabelled neighbouring cells. Finally, we investigated if QDs can be transferred via cell-cell fusion, and if the QDs themselves have any impact on the extent of cell fusion.

## Methods

### Ethics statement

The only animal work in this study involved the use of mid-gestation mouse embryos. Dams and embryos were sacrificed using schedule 1 procedures, which do not require ethical approval or a UK Home Office animal licence. Dams were culled using CO_2_ incubation followed by cervical dislocation. Embryos were dissected out from uterine horns and decapitated, and the kidney rudiments were dissected. These procedures were carried out at the University of Liverpool's designated animal facility.

### Cell culture

The E14.1 mouse ESC line was originally derived from the inbred mouse strain 129/Ola in 1985 by Martin Hooper in Edinburgh, Scotland, UK. The E14.1a ESC line used here was obtained from the Mark Boyd Laboratory at the University of Liverpool. The cells were cultured in advanced high glucose DMEM (Invitrogen, UK) supplemented with 2% FCS (PAA laboratories, UK), 2 mM L-glutamine (Sigma, UK) and 0.01% (v/v) 50 mM 2-mercaptoethanol (Invitrogen) on plastic tissue culture dishes (Nunc, Denmark) coated with 0.1% (w/v) gelatine (Sigma). Mouse KSCs were generated by Cristina Fuente Mora from mouse neonatal kidneys in our lab [16]. To generate EGFP^+^ cells (KSC-GFP), KSC cells were transduced with an EGFP-expressing lentivirus under the control of the spleen focus-forming virus (SFFV) promoter, pseudo-coated with a vesicular-stomatitis-virus glycoprotein (VSV-G) envelope. HEK293T cells were obtained from ATCC (Middlesex, UK). KSC and HEK293T cells were cultured in 10% (v/v) FCS DMEM medium supplemented with 2 mM L-glutamine. Both cell types were passaged every 3 days by trypsinisation and were cultured at 37°C in a humidified atmosphere containing 5% CO_2_.

### QD labelling

Cells were labelled with QDs (Invitrogen, Qtracker® Cell Labelling Kit, Q25021MP) according to the manufacturer's instructions. Briefly, QDs were mixed with 200 µl complete culture medium to give a final concentration of 10 nM and applied to 1×10^6^ cells in suspension. After 60 min incubation at 37°C and 5% CO_2_, the cells were washed 4X with complete growth medium and either cultured as usual or used for co-culture with unlabelled KSC-GFP cells in 2D culture (*in vitro* studies), or alternatively, co-cultured with unlabelled mouse E13.5 kidney rudiment cells (*ex vivo* 3D study). Where required, mitomycin C was used to block cell divisions; ESC were treated with 5 µg/ml mitomycin C (Sigma) for 2 h, and KSC were treated with 20 µg/ml mitomycin C for 3 h, following which, cells were washed 3X in PBS and subcultured as usual.

### Cell viability and growth

Following QD-labelling, the viability of cells was determined by trypan blue exclusion assay. Briefly, 0.01 ml of a 0.4% solution of trypan blue in PBS was added to 0.01 ml of cell suspension and incubated for 3 mins. The number of viable (unstained) and non-viable (blue) cells were counted using a haemocytometer and cell viability (%) was calculated as follows: total number of viable cells/total number of cells ×100. ESC and KSC population growth was assessed as follows: a total number of 15×10^3^ cells were plated at day 0 and the total numbers present at days 1, 2 and 3 were determined using a haemocytometer, following which population growth curves were constructed.

### Embryoid body (EB) formation

Following QD labelling, EBs were generated by culturing ESC in non-adherent petri dishes (Corning, UK). For endodermal differentiation, ESCs were cultured in high glucose DMEM (Invitrogen) supplemented with 10% (v/v) FCS (PAA laboratories), 2 mM L-glutamine (Invitrogen) and 0.01% (v/v) 50 mM 2-mercaptoethanol (Invitrogen). For mesodermal differentiation, ESCs were cultured in IMDM (Invitrogen) supplemented with 15% (v/v) FCS, 2 mM L-glutamine (Invitrogen), 0.15% (v/v) 100 mM monothioglycerol (Sigma), 1% 100X insulin-transferin-selenium (Sigma) and 0.1% (v/v) 500 mM ascorbic acid (Sigma). Both types of EB were collected for qPCR analysis following 4 and 7 days in culture.

### qPCR

Total RNA was extracted from cells and EBs using TRIzol reagent (Invitrogen) according to manufacturer's instructions. Following treatment with DNase1 (Promega, UK), the RNA was reverse transcribed using random hexamers (Thermo Scientific, Massachusetts) and Superscript III (Invitrogen). SYBR green-based qPCR was performed using KAPA Sybr Fast (Labtech, UK) and a RotorGene RG-3000 (Corbett Research, UK). The analysis was performed on 3 replicates, and the relative quantification of qPCR data for target gene expression in QD-labelled cells was normalised to endogenous reference gene expression (GAPDH), relative to untreated control cells [17]. Cycling parameters were: 1 cycle of 95°C/5 min, 35 cycles of 95°C/6 sec, 58°C/30 sec, 72°C/30 sec. Primers used are listed in Table 1.

**Table 1:** Primers used in this study.

Gene	Primers	Product size
***Brachyury***	F: *CATCGGAACAGCTCTCCAACCTAT*R: *GTGGGCTGGCGTTATGACTCA*	136 bp
***Gapdh***	F: *TGAAGCAGGCATCTGAGGG*R: *CGAAGGTGGAAGAGTGGGAG*	102 bp
***Gata6***	F: *CAAGATGAATGGCCTCAGCAG*R: *TGGTGGTGGTGTGACAGTTGG*	101 bp
***Oct4***	F: *TGGAGACTTTGCAGCCTGAG*R: *CTTCAGCAGCTTGGCAAACTG*	188 bp
***Pax6***	F: *GAGAAGAGAAGAGAAACTGAGGAACCAGA*R: *ATGGGTTGGCAAAGCACTGTACG*	201 bp
***Synaptopodin***	F: *GCCAGGGACCAGCCAGATA*R: *AGGAGCCCAGGCCTTCTCT*	73 bp
***Wt1***	F: *CCAGTGTAAAACTTGTCAGCGA*R: *TGGGATGCTGGACTGTCT*	234 bp

### Kidney rudiment culture

Chimeric kidney rudiments from E13.5 mouse embryos were cultured based on the method recently described by Unbekandt and Davies [18]. Single cell suspensions were produced from freshly isolated rudiments by trypsinisation and mechanical dissociation. QD-labelled KSC-GFP cells and unlabelled rudiment cells were counted using a haemocytometer and mixed in a 1 to 8 ratio. Cells were centrifuged at 1400× *g* for 3 min, the pellets transferred onto Isopore membrane filters (Millipore) on metal grids, and then cultured for up to 7 days in a humidified incubator at 37°C and 5% CO_2_. For the first 24 h of incubation the Y27632 Rho kinase (ROCK) inhibitor (Chemicon Int., Massachusetts) was applied at a final concentration of 5 µM. Medium was changed every second day. Samples were fixed in 4% (w/v) paraformaldehyde (PFA) for subsequent immunostaining.

### Immunostaining

For immunostaining, cells were fixed in 4% (w/v) PFA, washed 3X in PBS, and then incubated for 1 h at room temperature in blocking solution (10% (v/v) goat serum, 0.1% (w/v) Triton X100 in PBS). Primary antibodies (Table 2) were applied overnight at 4°C in 1% (v/v) goat serum, 0.1% (w/v) Triton X100 in PBS. The following day, cells were washed 3X in PBS and incubated with secondary antibodies (Table 2) in 1% (v/v) goat serum, 0.1% (w/v) Triton X in PBS for 2 h at room temperature in the dark, then washed 3X in PBS and incubated with 0. 05 ng/µl DAPI (4,6-diamino-2-phenylindole dihydrochloride, Invitrogen) for 5 min at room temperature in the dark. Cells were analysed either with Leica fluorescent microscope (Leica DMIL, Leica, Germany) or Leica confocal microscope (LEICA AOBS SP2).

**Table 2:** Primary and secondary antibodies.

	Name	Type	Concentration	Supplier
**Primary** **antibody**	**Oct4**	Mouse monoclonal IgG2B	1∶500	Santa Cruz, California,USA
	**Wt1**	Mouse monoclonal IgG1	1∶500	Upstate Massachusetts, USA
	**Synaptopodin**	Mouse monoclonal IgG1	1∶4	Acris, Germany
**Secondary** **antibody**	**Goat α Mouse**	IgG1 – 488	1∶500	Invitrogen
	**Goat α Mouse**	IgG2B – 488	1∶1000	Invitrogen

### QD exclusion assay and spectrophotometric analysis

ESC and KSC were labelled with QD as described above and plated onto 3.5 cm dishes (Nunc, Denmark). Cells were allowed to attach for 4 h, following which the culture medium was changed in order to remove any unattached cells. Medium was collected from cells after 48 h, and QDs were collected using a previously described method [12]. In brief, the culture medium was filtered through 0.22 µm syringe filter (Millipore, UK) and then centrifuged at 3500 g for 6 min. Pelleted QDs were either resuspended in 5 µl of component B from QD Tracker kit (Invitrogen), or were immediately resuspended in fresh culture medium and mixed with 1×10^6^ ESCs or KSCs. Following 1 h incubation with recovered QDs, cells were washed 4 X with culture medium, fixed in 2% (w/v) PFA and analysed by flow cytometry. For spectrophotometric analysis cells were labelled with QD as usual and washed 4X. Following each wash the medium was recovered. Medium was also recovered from QD-labelled cells following 48 h in culture; the medium was collected into tubes, centrifuged at 2000 rpm for 3 min and transferred into fresh tubes for spectrophotometric analysis. The analysis was performed using Cary Eclipse VARIAN Fluorescence Spectrophotometer (Agilent Technologies UK Limited, UK) using Cary Eclipse Software. The amount of QD in nM was established by comparison of sample fluorescence to standard curve readings.

### Cell lysis

10^6^ QD-labelled cells were cultured for 24 h after which they were trypsinised as described above and incubated in 10% (v/v) dimethyl sulfoxide (DMSO) in PBS for 3 h at 37°C. Resultant cell debris was vortexed for 30 sec and transferred into 3.5 cm dishes containing 5×10^4^ KSC-GFPs and cultured for 24 h. Cells were then washed 2 X with PBS, trypsinised and fixed in 2% (w/v) PFA for flow cytometry analysis.

### Cell fusion assay

The ability of QDs to increase the incidence of cell fusion was investigated using HEK 293T cells transfected with tandem dimer Tomato (tdTomato – HEK293-Tomato). The tdTomato plasmid was kindly provided by Dr Diana Moss (University of Liverpool). The cells were transfected using Lipofectamine 2000 (Invitrogen) after seeding for 1 day to obtain 90% confluency. Lipofectamine 2000 and DNA were diluted separately in Advanced DMEM (Invitrogen), incubated for 5 min, before mixing and incubating for a further 20 min at room temperature according to manufacturer's instructions. The mix was then added to 5×10^5^ cells for 24 h, after which the cells were washed with 1× PBS and trypsinised as described before and labelled with QDs. 5×10^4^ labelled or unlabelled cells were then co-cultured with 5×10^4^ KSC-GFP cells and their fusion determined after 24 h and 72 h culture using fluorescence microscopy and flow cytometry.

### Flow cytometry

QD-labelled cells were either fixed immediately after labelling (day 0) or cultured for up to three days. At specified time points the cells were trypsinised as usual and fixed in 2% (w/v) PFA and kept at 4°C for a maximum of 7 days until analysed with a flow cytometer (LSR II, BD Biosciences, Belgium). The data were analysed using BD FACSDiva software (BD Biosciences). For each analysis (3 replicates) the data from 10^4^ cells were gathered. The tdTomato emitted at 581 nm and QDs emitted at 655 nm; the compensation was set according to appropriate controls.

### Time lapse studies

ESC and KSC, with and without mitomycin C (MMC) treatment, were labelled with QD as described above and plated onto 3.5 cm dish (Greiner Bio One Ltd). The cells were imaged using a Zeiss LSM 510 confocal laser scanning system mounted on a Zeiss Axiovert 200 M (Carl Zeiss, Germany), with temperature controlled at 37°C and concentration of CO_2_ at 5.0%. For all acquisition settings, the main beam splitter was HFT 488/543. QDs were excited at 543 nm light and detected with a 560 nm longpass (LP) filter. Phase contrast images were recorded simultaneously with the red fluorescence in the transmission channel. Data capture was carried out every 10 min with Zeiss AIM software- (Zeiss, Germany) using the Auto-time series macro [19] and concatenated for each location. The obtained movies were then formatted using Pinnacle Studio software (Pinnacle Systems Ltd., UK).

### Statistical analysis

For all statistical data analyses, three independent replicates were used (n = 3). For image analysis, 6 different fields of view per sample were randomly selected. All data are shown as a mean +/− of standard error. Data sets were compared using Student's *t test* with p<0.05 considered as significant.

## Results and Discussion

### QDs do not affect the viability or growth rate of ESCs and KSCs

**Figure 1:**
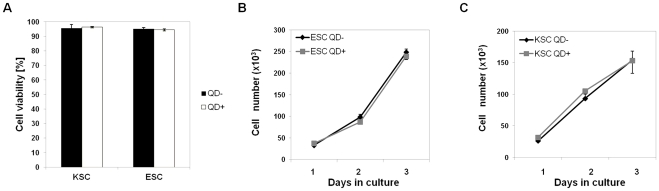
Cell viability and population growth following QD labelling. **A**) KSC and ESC viability measured by trypan blue exclusion from QD-labelled (QD^+^ - black bar) and unlabelled cells (QD^−^ - grey bar) immediately after QD labelling. **B**) Population growth curves for unlabelled control ESCs (ESC QD^−^) and QD-labelled ESCs (ESC QD^+^). **C**) Population growth curves for unlabelled control KSCs (KSC QD^−^) and QD-labelled KSCs (KSC QD^+^); n = 3 for each experiment.

To investigate the effect of QDs on the viability of ESCs and KSCs, both cell types were labelled with QDs for one hour, following which viability was determined by trypan blue exclusion. The results showed that QDs had no immediate effect on the viability of either ESCs or KSCs when compared to unlabelled controls (Figure 1A). To investigate if QDs had any effect on population growth, the number of ESCs and KSCs were counted over a 3 day period. QD labelling had no effect on the population growth of either stem cell type when compared to unlabelled controls (Figure 1B and C). It has been reported previously that QDs can be cytotoxic due to the release of Cd^2+^ ions from the CdSe or CdTe core of the QD [20]. However, the QDs used in this study were coated with a ZnS shell, which has been reported to prevent the release of Cd^2+^ ions thereby circumventing cytotoxic effects [6]. Thus, the lack of toxicity we demonstrate here for the ESC and KSC cells is consistent with other studies using ZnS-coated QDs on other cell types [11], [12], and so the previously reported cytotoxicity [15] is therefore likely to reflect the QD chemical composition or carrier vehicle (liposome-based transfection [15]) rather than being inherent to all QDs.

### QDs do not affect the differentiation potential of ESCs and KSCs

**Figure 2:**
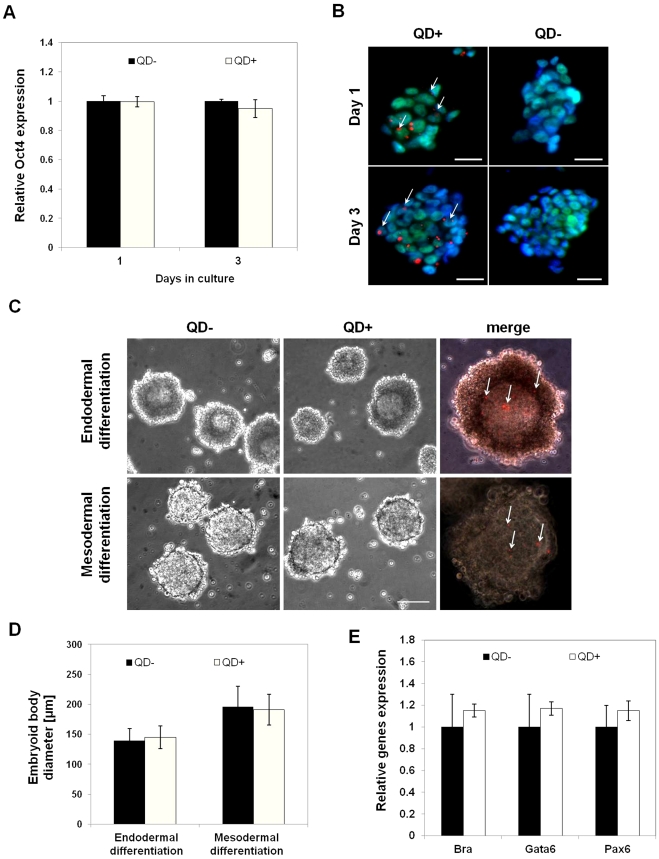
QDs do not affect the expression of pluripotency and lineage-specific markers in mouse ESCs. **A**) QPCR analysis of *Oct4* mRNA level in unlabelled (ESC QD^−^) and labelled (ESC QD^+^) after 1 and 3 days in culture. **B**) Immunofluorescent staining for Oct4 protein in both ESC QD^−^ and ESC QD^+^ cells showed nuclear localisation of Oct4 (green); white arrows in lower panel indicate Oct4^+^ QD^+^ cells. Nuclei are stained with DAPI (blue). Scale bar −50 µm. **C**) Top panel shows that following 4 days of culture under conditions that promote the differentiation of extra-embryonic endoderm, EBs derived from both control (QD^−^) and labelled (QD^+^) ESCs developed a thick layer of extra-embryonic endoderm at the EB periphery. Bottom panel shows that following 4 days of culture under conditions that promote mesoderm differentiation, EBs derived from both QD^−^ and QD^+^ ESCs developed a thin layer of outer extra-embryonic endoderm. Scale bar −100 µm. Images in the right-hand panel show the presence of QDs in 4 day EBs. **D**) QDs did not affect the size of the EBs under both endoderm- and mesoderm-promoting conditions. **E**) qPCR analysis of the endoderm-specific gene, *Gata6* mRNA, the mesoderm-specific gene, *brachyury* (Bra) and the ectoderm specific gene, *Pax6*, showed no significant difference in expression levels between EBs generated from QD^−^ ESC and those generated from QD^+^ ESC. The reference gene used for qPCR was *Gapdh*; n = 3 for each experiment.

**Figure 3:**
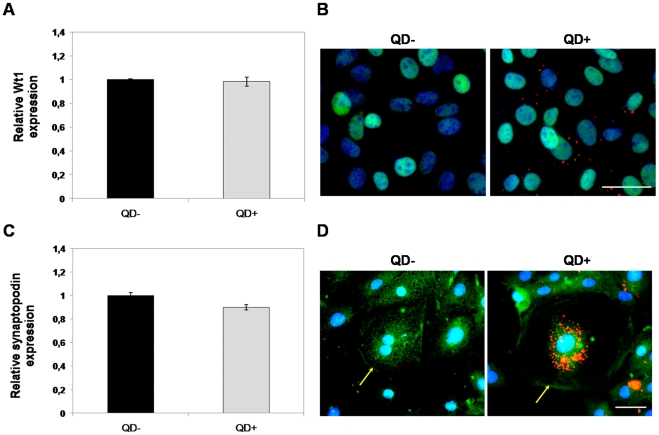
QDs do not affect the expression of lineage-specific markers in mouse KSCs. **A**) QPCR analysis of the KSC marker, *Wt1* mRNA, in unlabelled (QD^−^) and labelled KSCs (QD^+^) after 2 days in culture did not show any significant difference in expression levels. **B**) Immunofluorescent staining for Wt1 protein (green) showed no difference in expression between control and QD-labelled cells. Nuclei are stained with DAPI (blue). Scale bar −50 µm. **C**) QPCR analysis of the podocyte-specific marker, *synaptopodin*, mRNA in unlabelled (QD^−^) and labelled KSCs (QD^+^) after 2 days in culture did not show any significant difference in expression levels. **D**) Immunostaining for synaptopodin (green) showed nuclear and membrane localisation in both unlabelled (QD^−^) and labelled (QD^+^) podocyte-like cells (yellow arrow). Nuclei are stained with DAPI (blue). Scale bar −50 µm. The reference gene used for QPCR was *Gapdh*; n = 3 for each experiment.

Quantitative PCR (qPCR) analysis of ESCs on the first and third days following QD labelling showed that mRNA levels of the pluripotency transcription factor, Oct4, were not significantly different in labelled cells compared to controls (Figure 2A). Furthermore, the majority of QD-labelled cells displayed Oct4 immunoreactivity, confirming that the QDs do not appear to promote ESC differentiation when cultured under standard self-renewal conditions (Figure 2B). To investigate if QDs affected the ability of ESCs to differentiate, following QD-labelling, cells were cultured in suspension to promote the formation of embryoid bodies (EBs), using media optimized for either endoderm differentiation [21] or mesoderm differentiation [22]. After 4 days in the endoderm-promoting medium, both control and QD-labelled ESCs generated EBs with a thick outer layer of endoderm overlying a distinct basement membrane. Correspondingly, the mesoderm-promoting conditions resulted in QD-labelled EBs that were indistinguishable from controls, having only a thin ring of outer endoderm cells (Figure 2C). The diameters of the QD-labelled EBs was not significantly different from that of controls under either culture condition (Figure 2D). qPCR analysis of EBs at 7 days showed that expression levels of the endoderm-specific gene, *Gata6* [23], the mesoderm-specific gene, *brachyury* [24] and the ectoderm-specific gene, *Pax6* [25], were not significantly different in EBs generated from QD-labelled ESCs compared to controls (Figure 2E). To investigate the effect of QDs on KSC differentiation, qPCR analysis showed that mRNA levels of the *Wt1* KSC marker [16] were not significantly different between QD-labelled KSCs and controls (Figure 3A). Furthermore, the majority of QD-labelled cells displayed Wt1 immunoreactivity (Figure 3B). Additionally, mRNA levels (Figure 3C) and immunoreactivity (Figure 3D) of the podocyte-specific marker *synaptopodin* expressed by spontaneously differentiating KSC [16] were not significantly different in QD-labelled KSC compared to unlabelled controls. The increased number of QD aggregates in the podocyte-like cells compared to the KSCs was likely due to the fact that podocytes are terminally differentiated cells that do not proliferate (Figure 3D). Taken together, these results show that QDs do not appear to affect ESC or KSC potency. It has been reported previously that QDs inhibited the differentiation of MSCs to osteoblasts and chondrocytes [14], [15]. It should be noted that the QDs used in those studies were negatively charged and were delivered to the cells using liposome-based transfection [14], [15], while it has been shown recently that positively charged QDs, similar to those used in the current study, did not inhibit MSC osteogenesis [11]. It is therefore possible that in addition to any effects of QD surface chemistry [20], the delivery method might have the potential to affect stem cell differentiation.

### QDs have a high labelling efficiency but are depleted rapidly from ESCs and KSCs

**Figure 4:**
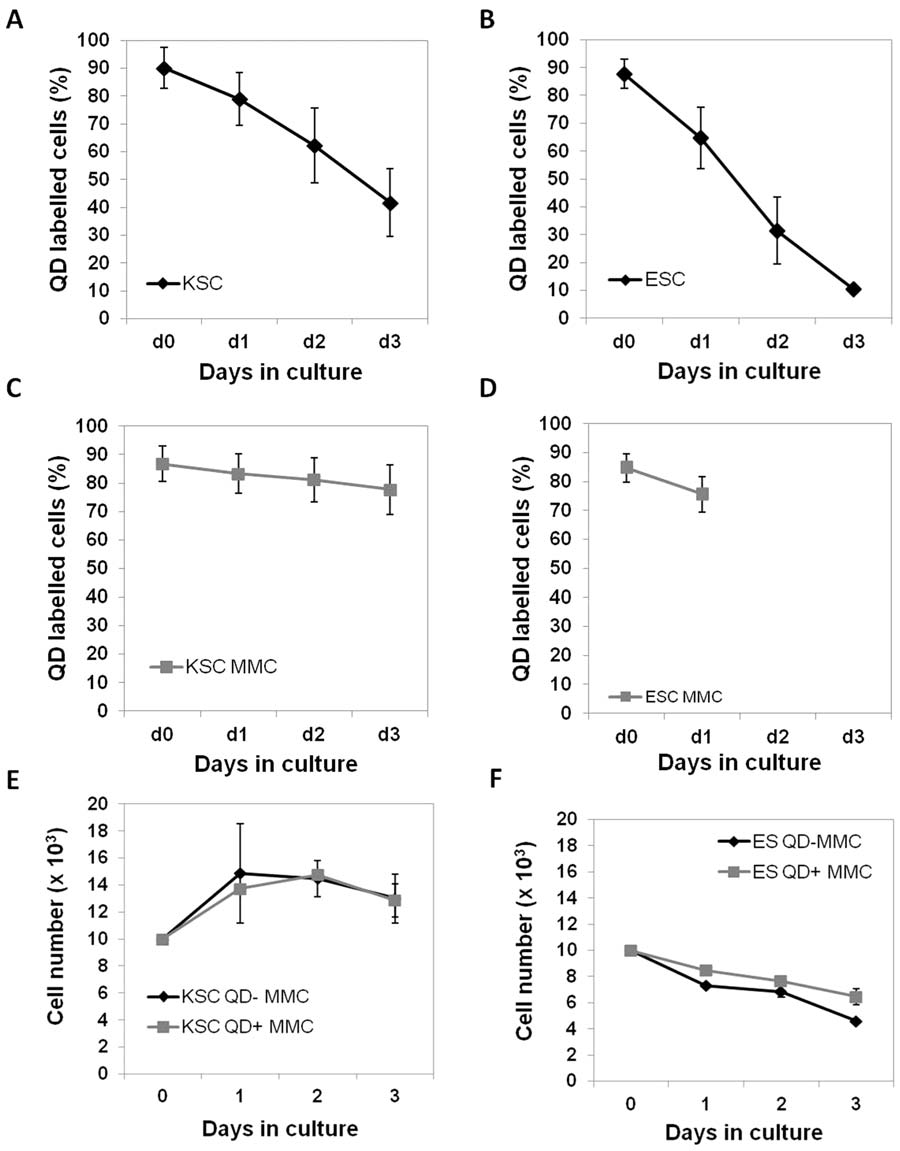
ESC and KSC do not excrete QD. **A–B**) Following 3 days in culture about 40% of KSCs were QD-labelled (A) and only 10% of ESC were QD-labelled (B). **C–D**) Treatment with mitomycin C (MMC) inhibited QD loss from KSC over a 3 day time course (C) and from ESC over a 24 h time course (note that after 24 h, MMC-treated ESCs had undergone cell death and analysis at later time points was not possible) (D). **E–F**) The proliferation growth curve of QD-labelled and unlabelled KSC (E) and ESC (F) following treatment with MMC.

In order for QDs to be useful for cell tracking, it is necessary that a reasonable proportion of cells within the population retain their label throughout the time course of the experiment. To investigate if ESCs and KSCs retained the QDs, the cells were imaged daily for a period of 3 days. At day 0 (2 hours following labeling), the majority of ESCs and KSCs were labelled, and it was found that the QDs were localised to a few large aggregates in close proximity to the nucleus (Figure S1). The staining pattern of the QDs suggested that they were localised to late endosomes and lysosomes, as previously described [12]. By day 3, the proportion of labelled cells in both cell populations was noticeably reduced; FACS analysis showed that about 85% of both stem cell types were labelled at day 0, but by day 3, the percentage of labelled ESCs and KSCs had fallen to 10% and 40%, respectively (Figure 4A and B).

**Figure 5:**
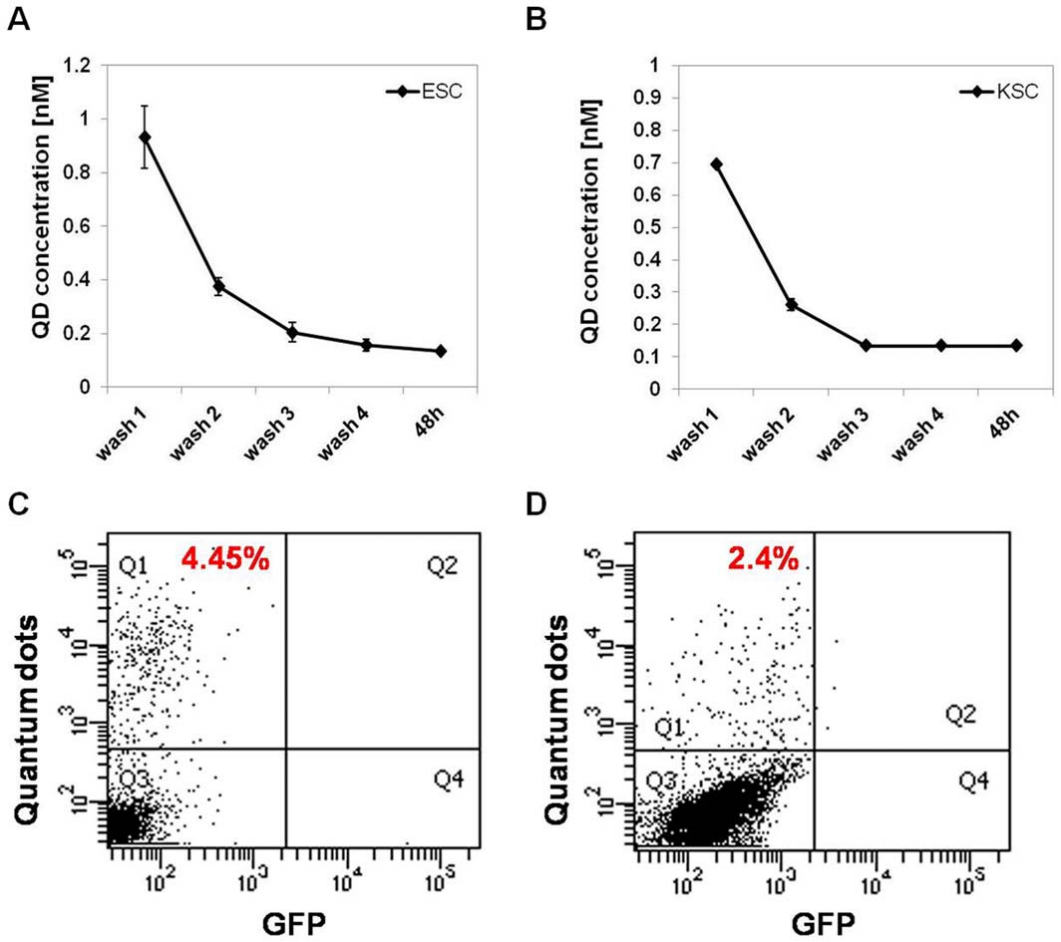
Flow cytometric analysis shows that QDs excluded from cells display a poor re-labelling efficiency. **A–B**) Fluorometer analysis of the medium collected following each of the 4 washes immediately after QD labelling, and of medium collected from KSC (A) and ESC (B) following 48 h of culture. Note that the medium was not filtered, only centrifuged in order to eliminate cells from the suspension; n = 3 for each timepoint. **C**) QDs (0.134 nM) collected from ESC culture medium following 48 h of initial labelling, labelled 4.45+/−0.12% of a fresh ESC population. **D**) QDs (0.134 nM) collected from KSC culture medium following 48 h of initial labelling, labelled 2.4+/−0.3% of a fresh KSC population; n = 3 for each experiment.

To determine if QD depletion was due to cell division, mitomycin C was used to block cell proliferation and the number of QD-labelled cells was determined using flow cytometry. If the decrease in the number of QD-labelled cells was entirely due to signal dilution following cell division, it would be expected that mitomycin C treatment should prevent signal loss. In the presence of mitomycin C, the percentage of QD-labelled KSCs did not significantly decrease between days 1 and 3, leading to the conclusion that cell division was the main reason for the reduction in the number of labelled KSCs (Figure 4C). In contrast, the mitomycin C had a negative impact on ESC resulting in excessive cell death (Figure 5F, Movie S4); therefore, FACS analysis beyond day 1 was not possible (Figure 4D). To confirm that QD loss over time is due to cell division rather than excretion, as suggested by Pi et al. [12], we have investigated the amount of QD in the medium after 2 days in culture using fluorometry. We found that the amount of QD present in the medium after 2 days in culture was negligible (<0.15 nM), suggesting that the QD are not released from cells (Figure 5A and 5B). In order to confirm the above results, time lapse studies were performed of QD-labelled ESC and KSC, both with and without treatment with mitomycin C (MMC). They revealed that over the time-course of the experiment, neither KSC, nor ESC, appeared to excrete QDs (Movie S1, S2 and S3). Moreover, upon MMC-induced cell death of ESC, we did not observe any QD release (Movie S4). Taken together, these results show that QDs are diluted in ESCs and KSCs by cell division and not by QD excretion from cells. However, due to the fact that this depletion is quite rapid, QDs are only suitable for relatively short-term tracking.

### QDs do not readily transfer to neighbouring cells in 2D co-culture

For cell tracking experiments, it is important to establish if any QDs could be transferred from labelled cells to unlabelled neighbouring cells, as this could lead to false results. Although the medium collected from labelled cells after 48 h of culture contains very low amounts of QDs (<0.15 nM) (Figure 5A, 5B), we nevertheless collected this medium from QD-labelled ESC and KSC to ascertain if any QDs present were able to label a fresh batch of ESCs and KSCs, respectively. Flow cytometry showed that QD-containing medium collected from ESCs labelled ∼4% of cells, while medium collected from KSCs labelled only ∼2% of cells (Figure 5C and 5D). To investigate if the reason for poor re-labelling efficiency was that the coating peptide required for delivering the QDs inside the cells had been modified or removed within the intracellular environment, the experiment was repeated, but prior to re-labelling, QDs collected from the culture medium were incubated in fresh coating peptide. It was found that the re-application of coating peptide did not significantly affect the results (data not shown).

**Figure 6:**
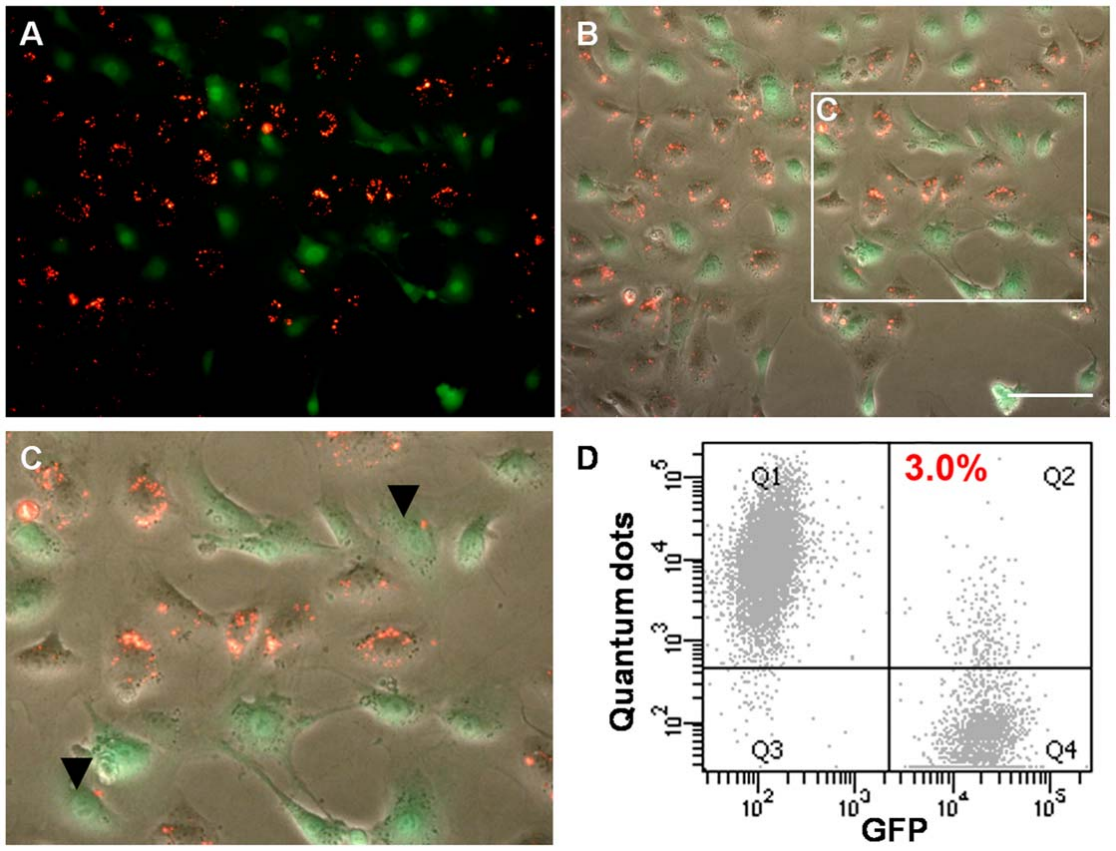
Co-culture of QD-labelled KSC and KSC-GFP cells showed minimal QD transfer. **A–C**) Following 24 h co-culture, very few GFP^+^ cells were labelled with QDs (arrowheads in **C**); **A**) QD^+^ cells (red); GFP^+^ cells (green); **B**) phase contrast and fluorescence overlay; **C**) Zoom of boxed area in B. **D**) Flow cytometric analysis shows that following 24 h of co-culture, only 3% (+/−0.09%) of cells were GFP^+^QD^+^; n = 3.

Our findings are markedly different from those described in a recent report by Pi et al., where it was shown that QDs excreted by ESCs could re-label >20% of a fresh ESC population [12]. However, in the Pi et al. study, only one wash step was used to remove the QDs from the cell suspension following re-labelling, whereas we routinely perform four washes. It is thus possible that the excess of QD aggregates in the Pi et al. study might have remained stuck to the outside of the cells, leading to false positive results when the cells were analysed using flow cytometry [12]. To investigate if QDs could be transferred to neighbouring cells under direct co-culture conditions, QD-labelled cells were mixed with a population of KSCs that constitutively expressed GFP (KSC-GFP cells). Following 24 h of co-culture, very few GFP+ cells contained QDs (Figure 6A, B and C) and flow cytometric analysis showed that the percentage of QD-labelled GFP+ cells was <4% (Figure 6D). Our findings contrast with those of Ranjbarvarizi et al, who showed 100% transfer efficiency of QDs from labelled cells derived from umbilical cord blood and bone marrow to neighbouring cells in co-culture experiments [11]. It has been reported that the behaviour of QDs inside cells can vary depending on the size of the QDs and their surface chemistry [26]. However, the QDs used in the current study were coated with the same positively charged peptides as those used by Ranjbarvarizi et al. [11]. Furthermore, although the QDs used here (QD 655 nm) were of a different size, this is unlikely to account for the differences in results, as Ranjbarvarizi et al. observed QD transfer to unlabelled cells when both smaller (585 nm) and larger (800 nm) QDs were used. Given that human umbilical cord blood-derived CD34+ cells, such as those used by Ranjbarvarizi et al. [11], display a high incidence of cell-cell fusion in 2D culture [27], then cell-specific fusion may account for the differing observations. Consistent with this notion, it has recently been shown that QDs did not transfer to neighbouring human bone marrow-derived MSCs [10].

### In *ex vivo* transplantation studies, the extent of QD transfer from grafted cells to host cells is negligible

**Figure 7:**
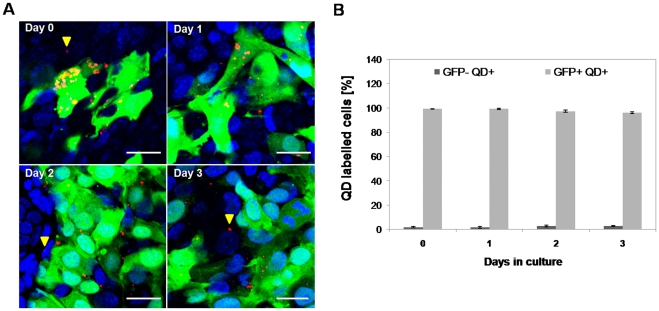
QD transfer from KSC-GFP cells into mouse foetal kidney cells in 3D ex vivo co-culture is negligible. **A**) Confocal microscopy of chimeric kidney rudiments shows that few host cells become labelled with QDs over a 3 day time course; (DAPI, blue; GFP, green; QDs, red). Note that in most fields of view, no GFP^−^ QD^+^ cells were present. The presented images have been selected to show rare GFP^−^ QD^+^ cells. Scale bar, 10 µm. **B**) Statistical analysis of QD transfer shows no significant difference in numbers of GFP^−^QD^+^ cells from day 0 (1.8+/−0.5%) and day 3 (2.5+/−0.4%) of culture; p>0.1 (n = 3).

The low level of QD transfer we observed in 2D KSC co-culture could be due to the fact that under these conditions there is only a limited degree of cell-cell contact between the QD-labelled and unlabelled GFP+ cells (Figure 6A and B). It was therefore important to determine the degree of QD transfer under conditions where the QD-labelled cells are completely surrounded by unlabelled host cells using organ culture. To this end, KSC-GFP cells were labelled with QDs, and mixed with freshly isolated mouse foetal kidney cells in order to form a chimeric rudiment [18]. The percentage of host rudiment cells that became labelled with QDs was determined daily over a 3 day culture period. The results showed that at day 0, the percentage of QD-labelled host cells was 1.8+/−0.5%, and importantly, only 2.5+/−0.4% of host cells were labelled by day 3 (Figure 7). Furthermore, the percentage of QD-labelled KSC-GFP cells at day 0 was 99+/−0.1%, and remained at 96+/−0.6% by day 3 (Figure 7). This result was dramatically different to that observed in 2D culture, where it was observed that only 40% of KSCs remained labelled with QDs following a 3 day culture period. The most likely explanation is that the proliferation rate of the KSCs is much slower within the kidney rudiment than in 2D culture.

### QDs released following cell death are not readily transferred to neighbouring cells

**Figure 8:**
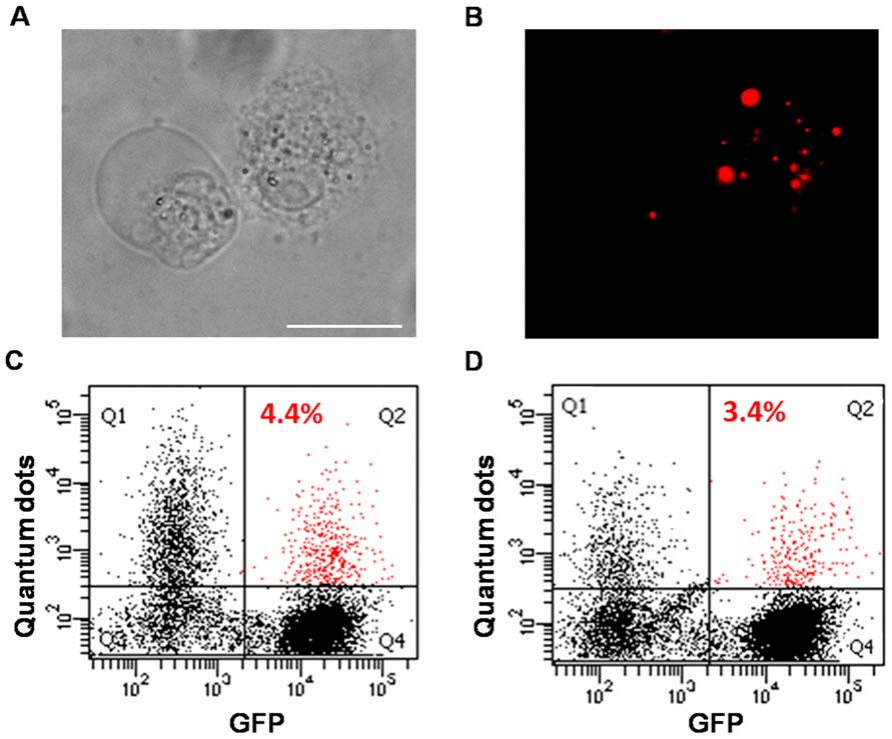
QDs released following cell death are not readily transferred to neighbouring cells. (**A, B**) Phase contrast (A) and fluorescent (B) image of QD-labelled KSC following treatment with DMSO. (**C, D**) Flow cytometric analysis showed that relatively few QDs released by dead cells were taken up by GFP-labelled KSC; QD from ESCs showed an average uptake of 4.4+/−3.3% (n = 3) (C) and QDs from KSC, 3.4+/−1.2% (n = 3) (D). Scale bars, 20 µm.

A potential problem with most of the commonly used labelling methods for cell tracking is that if the grafted cell dies, the label could be taken up by neighbouring host cells, leading to false positive results. The time lapse studies of ESC MMC treated cells showed cell death (Movie S4) and lack of QD release to medium; however these cells are not in close proximity to healthy cells. Therefore to investigate if QDs released from dead cells can be transferred to neighbouring cells, QD-labelled ESCs and KSCs were induced to undergo cell death by incubation in 10% dimethyl sulfoxide (DMSO) for 3 hours at 37°C. DMSO increases the permeability of the cell and intracellular membranes [28], especially at high temperature, and has marked cytotoxic effects following 2–3 h incubation for both KSC (Figure 8A and B) and ESC (not shown). Following DMSO treatment, the resultant cell debris was then co-cultured with a population of KSC-GFP cells for 24 h. Flow cytometry showed that irrespective of whether the KSC-GFPs were cultured with cell debris derived from QD-labelled ESCs or KSCs, the percentage of KSC-GFPs that became QD-labelled was <5% (Figure 8C and D).

### QDs do not increase the degree of cell-cell fusion

**Figure 9:**
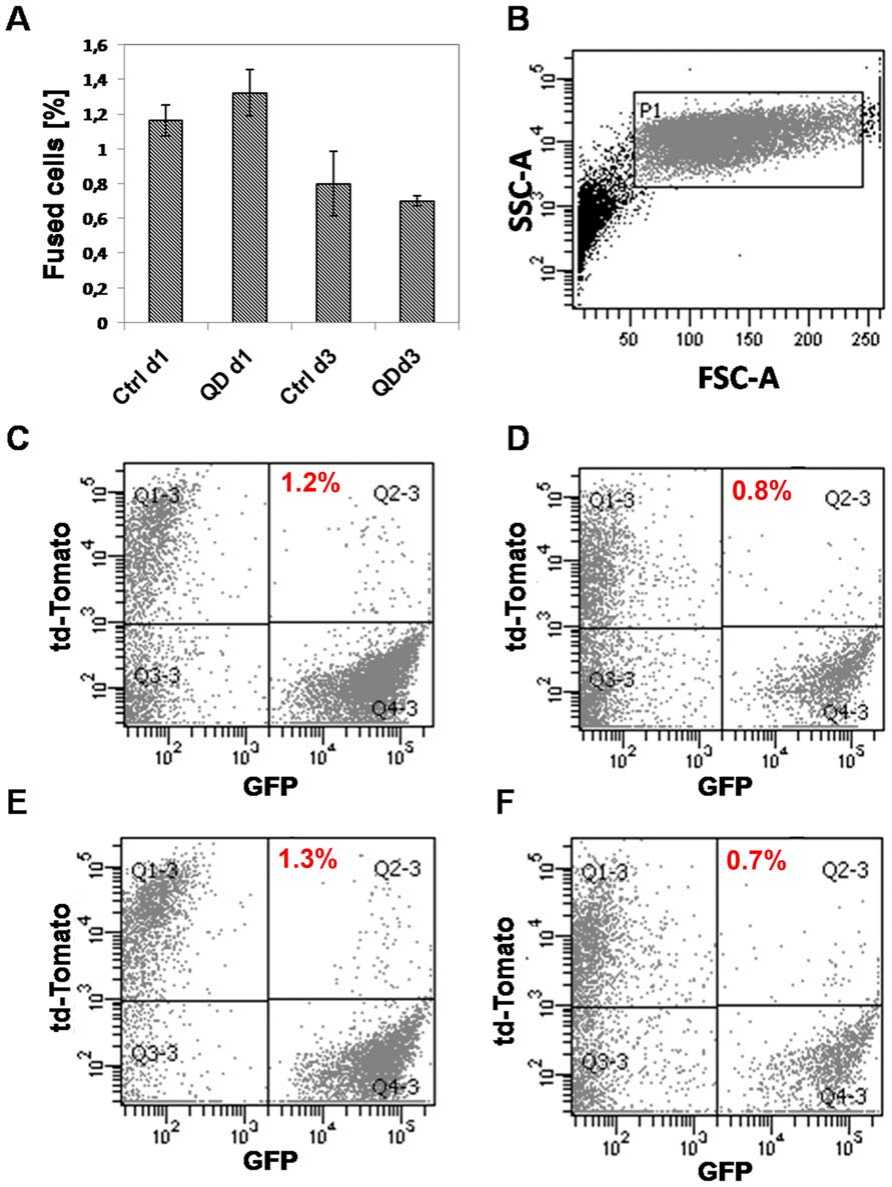
QDs do not increase cell-cell fusion. **A**) HEK-293T cells expressing tdTomato were labelled with QDs and co-cultured with KSC-GFP cells. Flow cytometric analysis showed that QDs did not increase cell-cell fusion over a 3 day time course. **B**) Flow cytometry data were collected for 1×10^4^ events for gated population, P1. **C–F**) Representative flow cytometry graphs showing the percentage of fused cells in controls (QD^−^) at day 1 (1.2+/−0.1%; C) and day 3 (0.8+/−0.2%; D) of culture; and in QD-labelled cells (QD^+^) at day 1 (1.3+/−0.1%; E) and day 3 (0.7+/−0.03%; F).

It has been well-established that some cell types can fuse with host cells following transplantation [29], [30], leading to false positive results. It is clear that the extent of fusion is dependent on a number of factors, including the type of cell that is transplanted, the target organ, and the condition of the host tissue. However, to our knowledge, the effect of the labelling reagent itself on cell fusion has not previously been studied. To investigate if QD-labelling increases the incidence of cell-cell fusion, human embryonic kidney (HEK293) cells expressing the tdTomato fluorescent protein (HEK293-tdTomato), were labelled with QDs and co-cultured with KSC-GFP cells. Controls consisted of co-cultures of unlabelled HEK293-tdTomato cells and KSC-GFP cells. The extent of fusion was investigated using flow cytometry following 24 h and 72 h of co-culture by determining the percentage of cells displaying both red (tdTomato) and green (GFP) fluorescence. It was found that the extent of cell-cell fusion in controls was negligible (<2%), and did not increase following QD labelling of the HEK293-tdTomato cells (Figure 9). Therefore it can be concluded that labelling with QDs does not affect cell fusion.

### Conclusion

In this study we have investigated the feasibility of using QDs to track mouse ESCs and KSCs. We found that QDs had a high labelling efficiency and had no effect on the viability or differentiation potential of ESCs and KSCs, but were rapidly depleted from both stem cell types when cultured under self-renewal conditions in 2D culture, indicating that they are only suitable for short-term tracking in rapidly proliferating cells. In contrast, when QD-labelled cells were cultured in 3D organ culture, conditions in which the stem cells are expected to differentiate, the degree of QD depletion was minimal; this was likely due to the lower proliferation rate of the cells following differentiation. Cell division was the main cause of QD depletion in KSCs and ESCs. QDs were not released into the extracellular environment through excretion, or due to cell death, and were not readily transferred to neighbouring cells. Furthermore, the QDs had no effect on the incidence of fusion. Taken together, our results show that the QDs used in this study (655 nm CdSe/ZnS dots coated with positively charged peptides) are suitable for short-term tracking of mouse ESCs and KSCs. However, given that QD behaviour can vary depending on size and surface chemistry, accompanied with the fact that some cell types, such as CD34+ cells, have a high propensity for cell-cell fusion, it is important to establish the effect of QDs on cell behaviour and the extent of QD transfer for each particular QD and stem cell type.

## Supporting Information

Figure S1 **Photomicrograph of KSC and ESC labelled with QD.**
(TIF)

Movie S1 **24 h time lapse confocal microscopy showing the behaviour of QD-labelled KSC in culture.** White arrows indicate some dividing cells. Proliferating cells round up before dividing to generate two daughter cells, all of which were labelled with QDs.
(AVI)

Movie S2 **24 h time lapse confocal microscopy showing the behaviour of QD-labelled KSC following treatment with mitomycin C.** These cells do not divide, only migrate around the dish. All cells remain labelled with QDs.
(AVI)

Movie S3 **24 h time lapse confocal microscopy showing the behaviour of QD-labelled ESC in culture.** The white arrow indicates an ESC undergoing cell division. The daughter cells later combine with a larger colony. It is clear that not all cells are labelled at the end of the 24 h time course. The green arrows indicate migrating MEF cells.
(AVI)

Movie S4 **24 h time lapse confocal microscopy showing the behaviour of QD-labelled ESC following treatment with mitomycin C.** These ESC do not divide and many of them are already dead at the start of the time course (∼7 h post plating). Yellow arrows indicate dying cells. In most cases the dead cells appear to retain their QDs.
(AVI)
