# Supplementary figures and images for: Quantum Dots Do Not Affect the Behaviour of Mouse Embryonic Stem Cells and Kidney Stem Cells and Are Suitable for Short-Term Tracking
Source: PLoS One. 2012 Mar 5;7(3):e32650. doi: 10.1371/journal.pone.0032650 (PMC3293847; doi:10.1371/journal.pone.0032650)

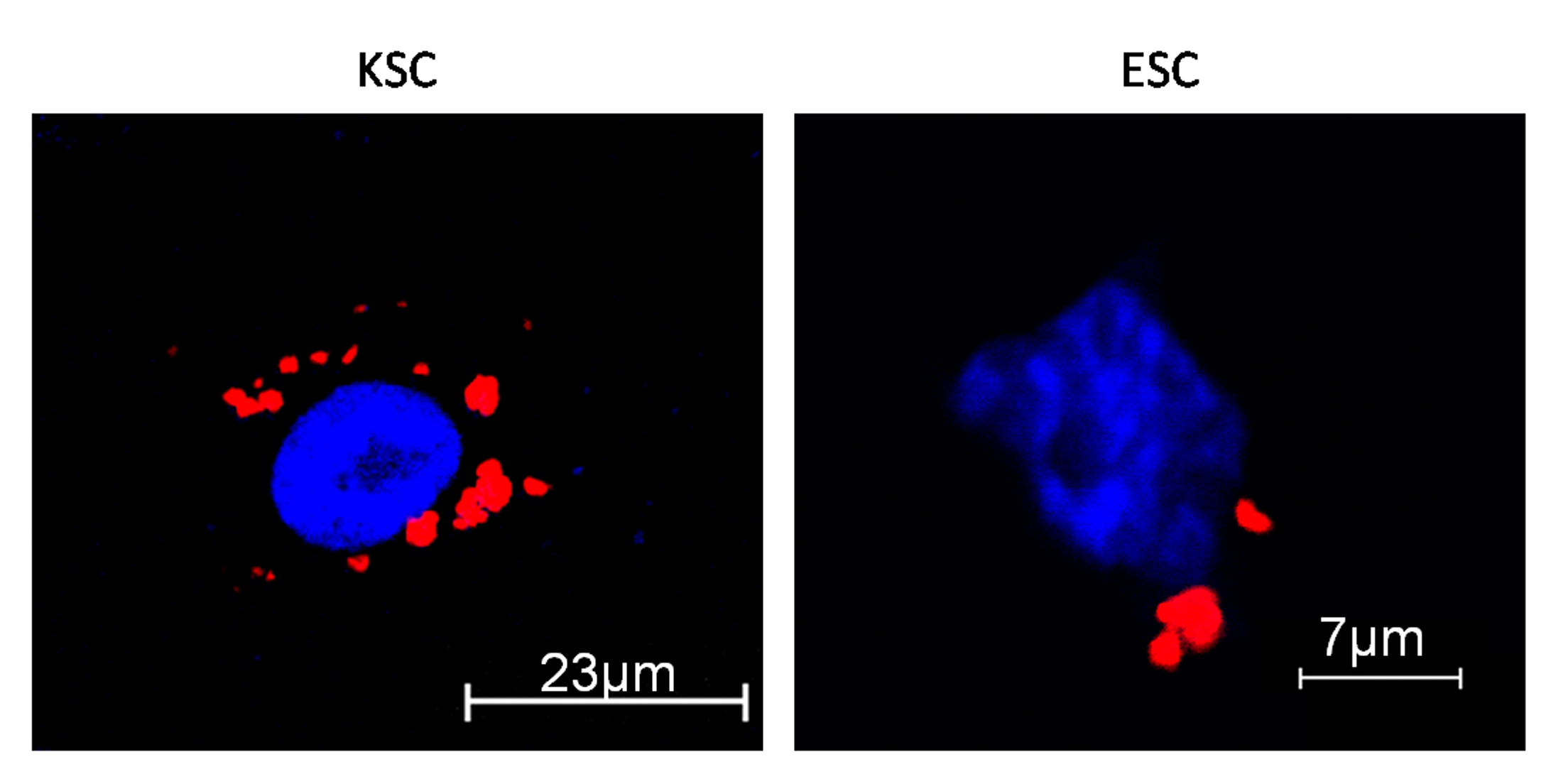

Supplement: Figure S1 — Photomicrograph of KSC and ESC labelled with QD. (TIF) [file pone.0032650.s001.tif]
